# Supplementary material for: Fecal microbiome composition and diversity of cryopreserved canine stool at different duration and storage conditions
Source: PLoS One. 2024 Feb 7;19(2):e0294730. doi: 10.1371/journal.pone.0294730 (PMC10849402; doi:10.1371/journal.pone.0294730)
Supplement: S1 Fig — The edges of the box represent the 25th and 75th percentiles. The whiskers represent the maximum and minimum values below and above the upper (75th percentile + IQR) and lower (and 25th percentile–IQR) fences, respectively. Individual black dots represent individual dogs. (PDF) [file pone.0294730.s002.pdf]

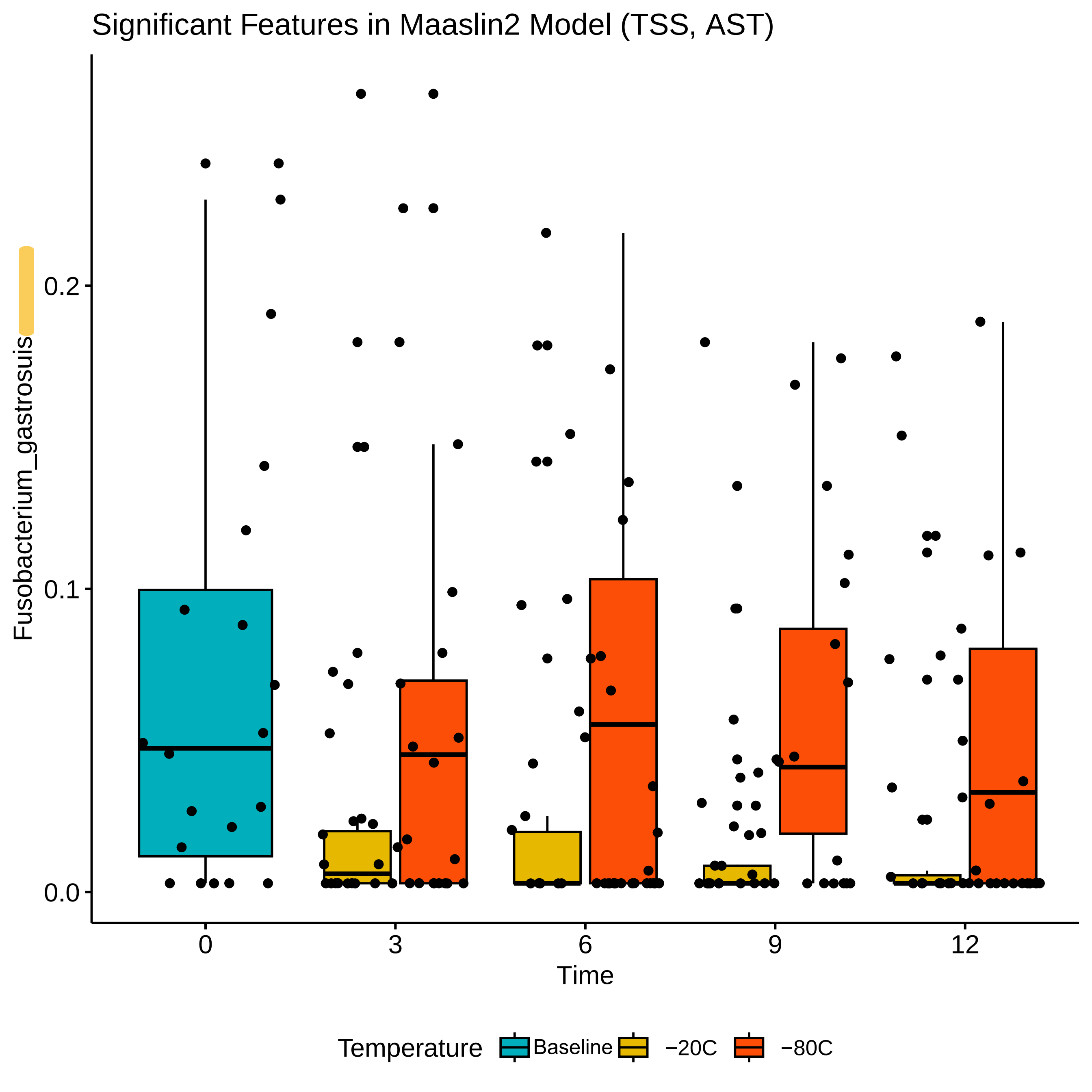

Significant Features in Maaslin2 Model (TSS, AST)
Fusobacterium_gastrosuis
0.2
0.1
0.0
0
3
6
9
12
Time
Temperature
Baseline
−20C
−80C

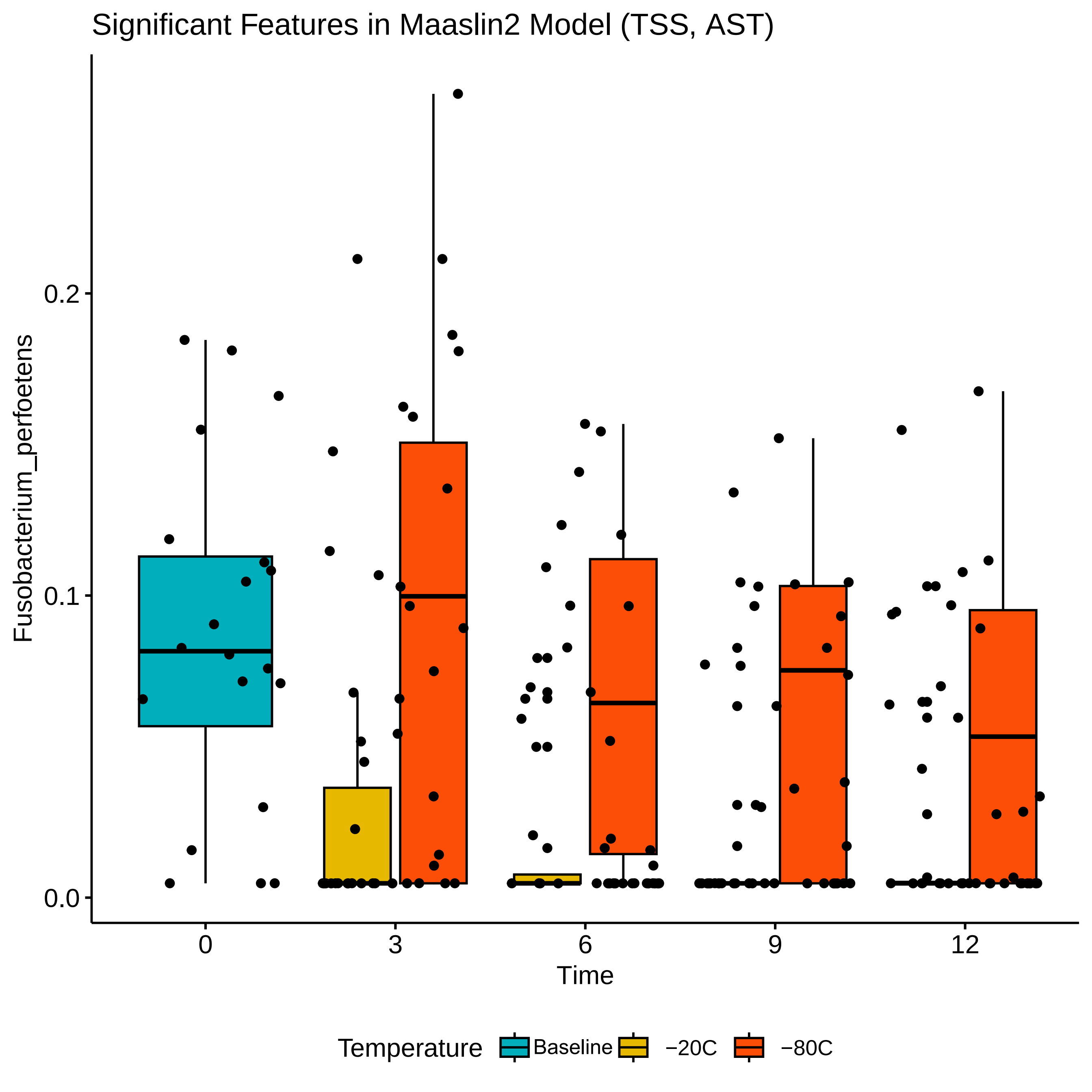
Significant Features in Maaslin2 Model (TSS, AST)
Fusobacterium_perfoetens
0.0
0.1
0.2
0
3
6
9
12
Time
Temperature
Baseline
−20C
−80C

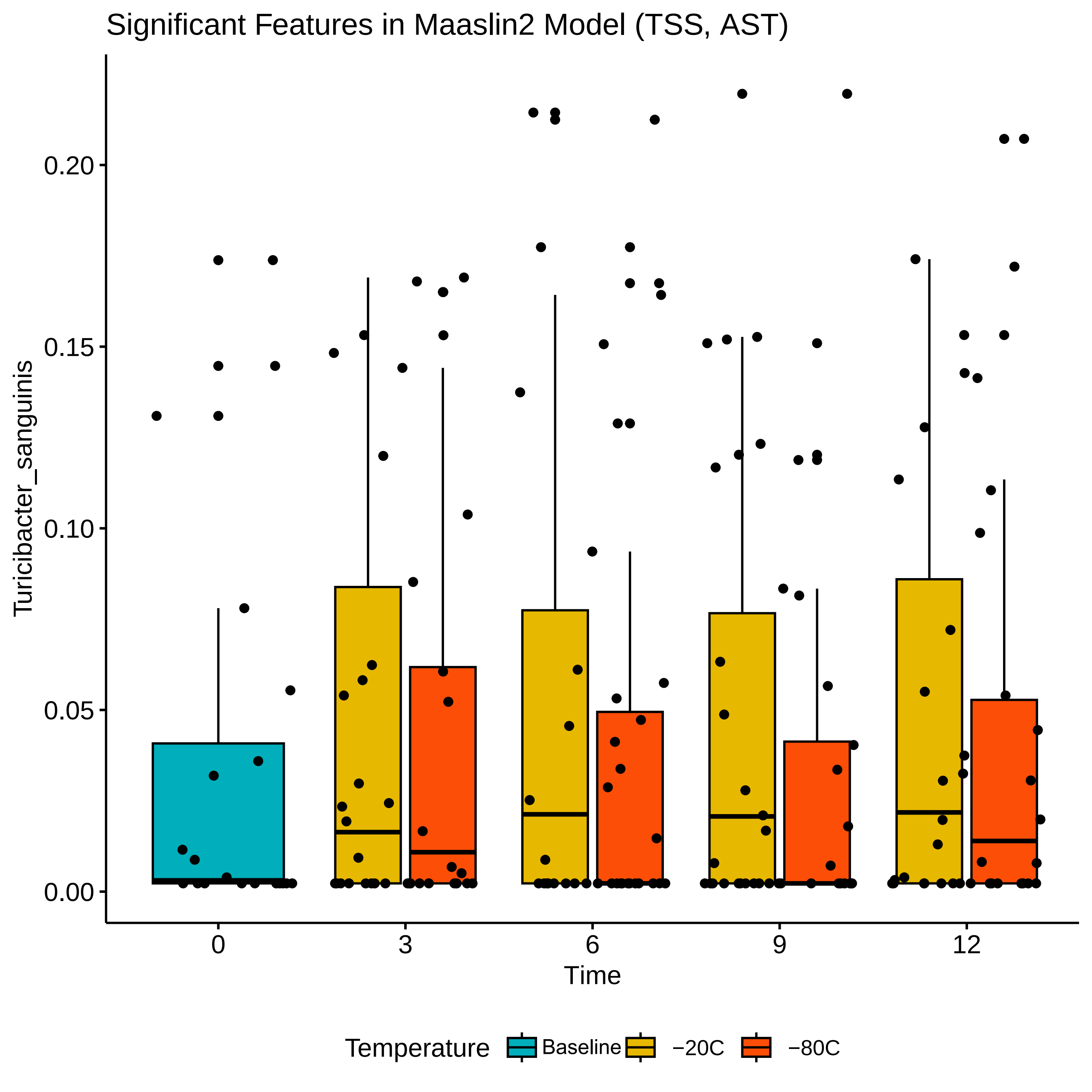

Significant Features in Maaslin2 Model (TSS, AST)
Turicibacter_sanguinis
0.20
0.15
0.10
0.05
0.00
0
3
6
9
12
Time
Temperature
Baseline
−20C
−80C

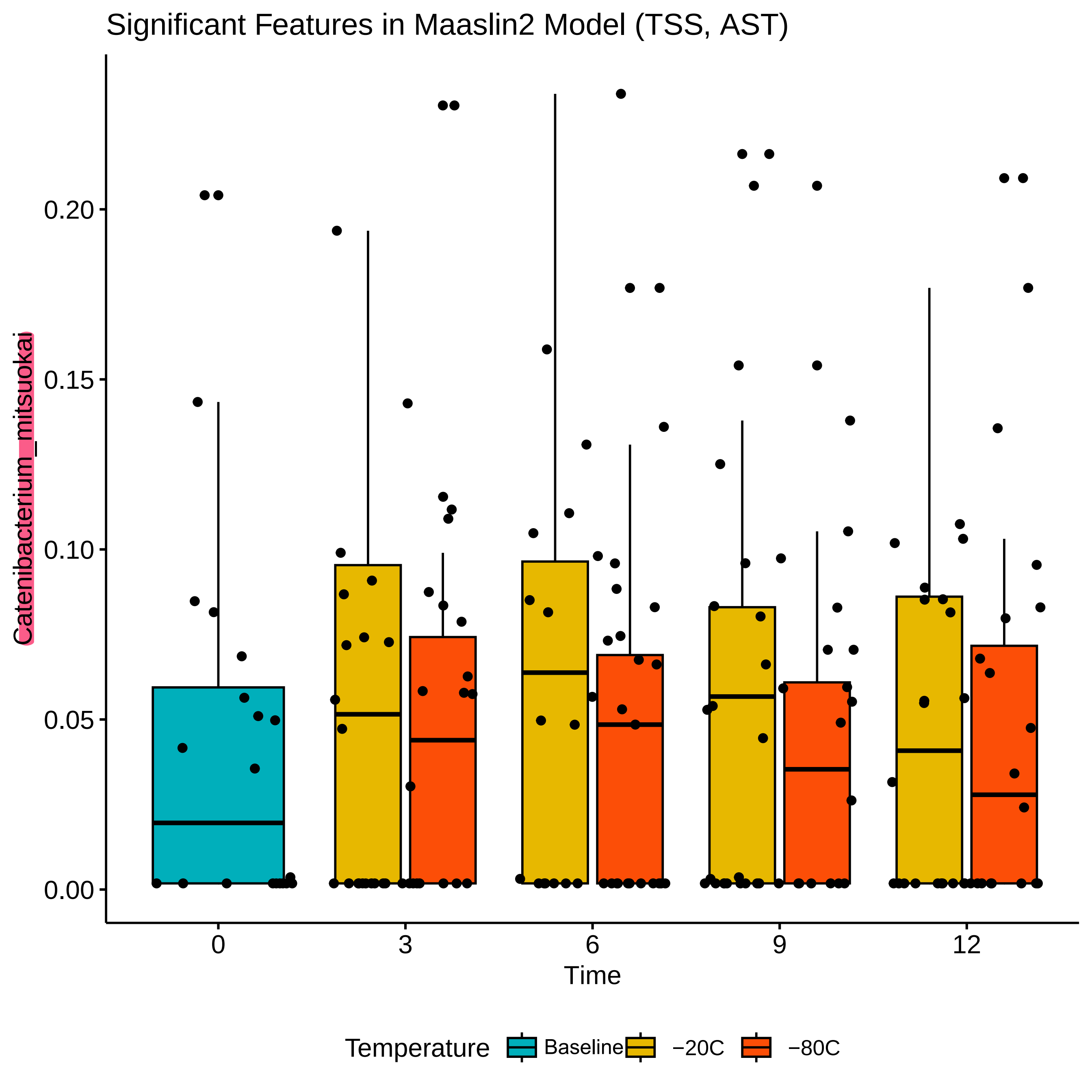

Significant Features in Maaslin2 Model (TSS, AST)
Catenibacterium_mitsuokai
0.00
0.05
0.10
0.15
0.20
0
3
6
9
12
Time
Temperature
Baseline
−20C
−80C

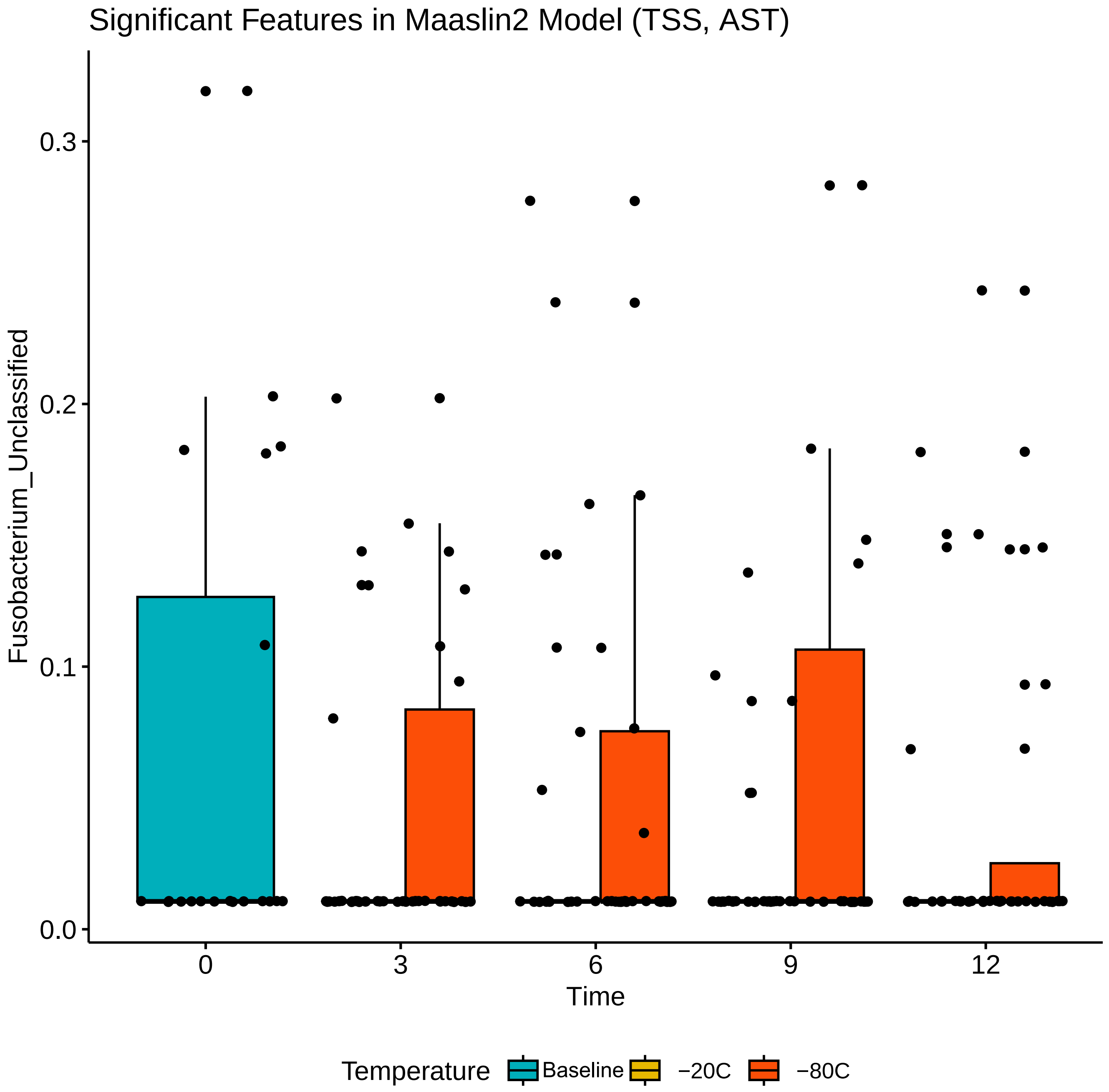
Significant Features in Maaslin2 Model (TSS, AST)
Fusobacterium_Unclassified
0.0
0.1
0.2
0.3
0
3
6
9
12
Time
Temperature
Baseline
−20C
−80C

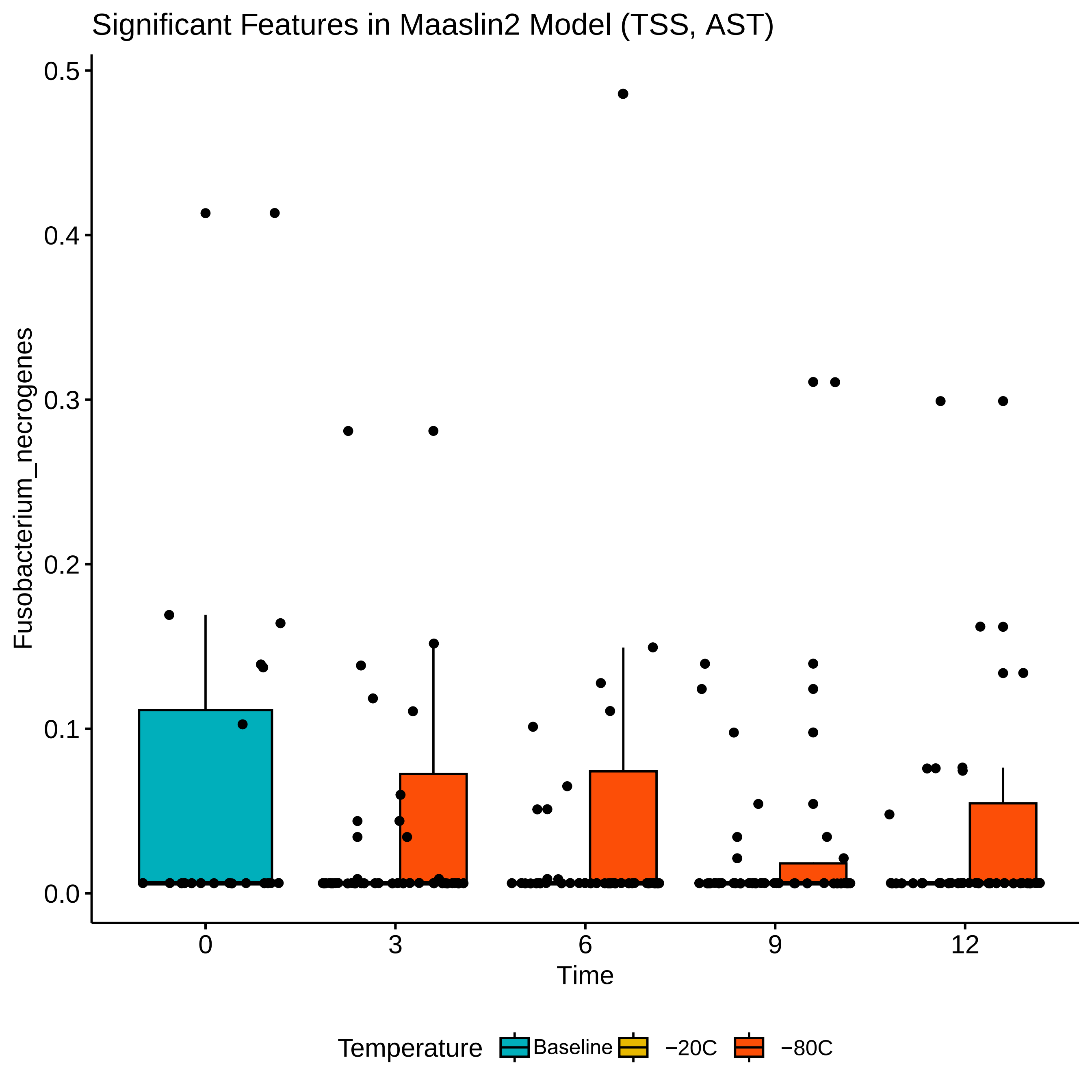

Significant Features in Maaslin2 Model (TSS, AST)
Fusobacterium_necrogenes
0.0
0.1
0.2
0.3
0.4
0.5
0
3
6
9
12
Time
Temperature
Baseline
−20C
−80C

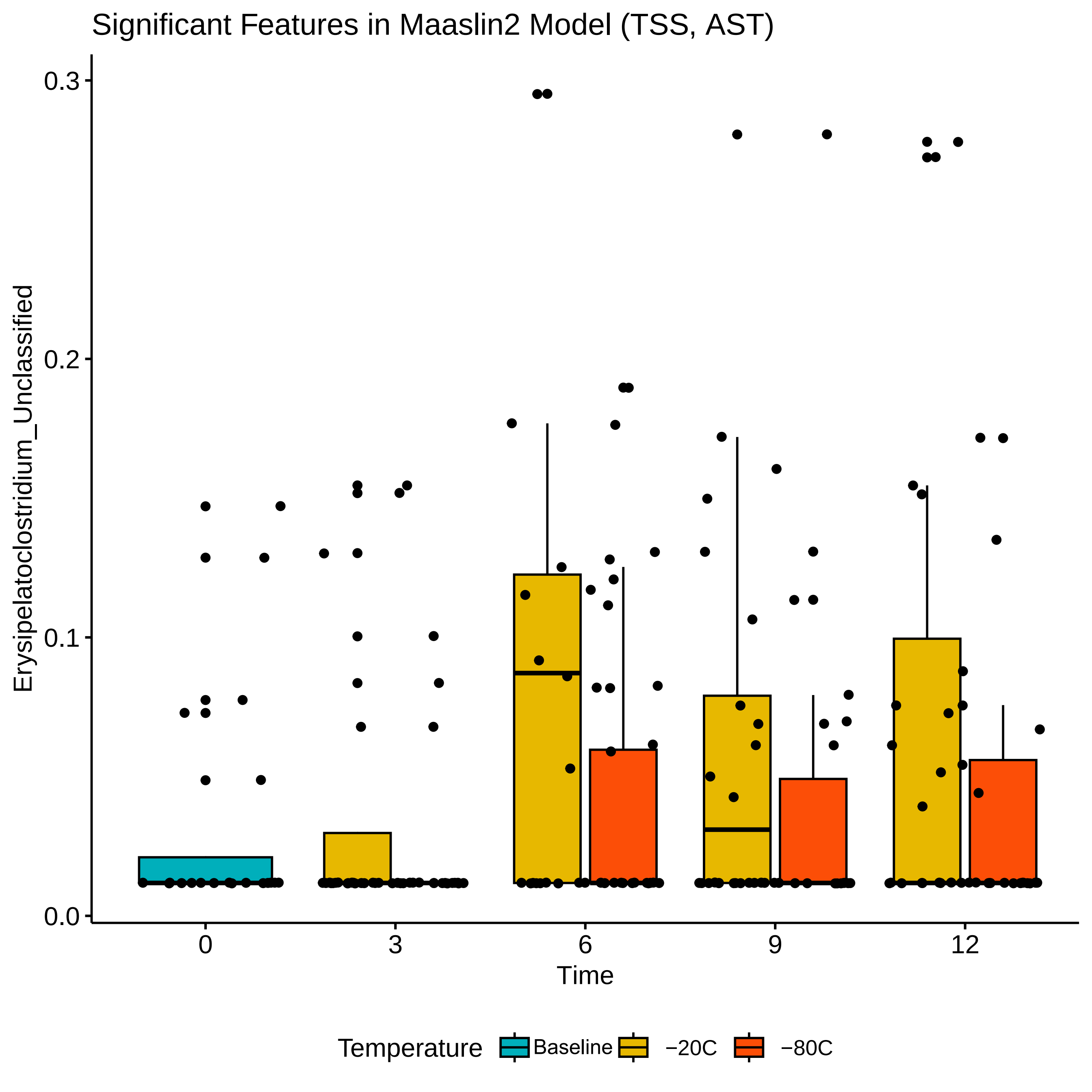
Significant Features in Maaslin2 Model (TSS, AST)
Erysipelatoclostridium_Unclassified
0.0
0.1
0.2
0.3
0
3
6
9
12
Time
Temperature
Baseline
−20C
−80C

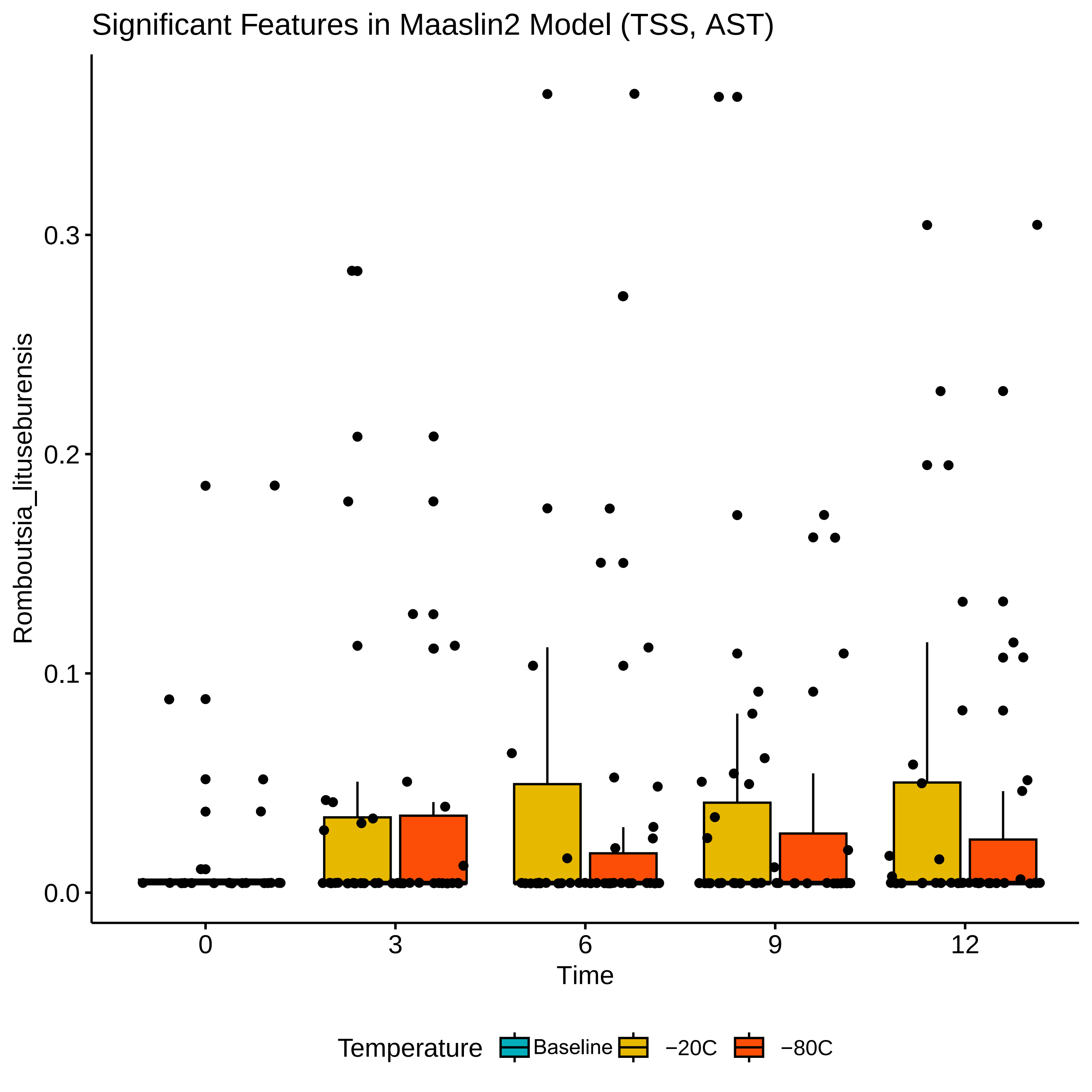

Significant Features in Maaslin2 Model (TSS, AST)
Rombousia_lituseburensis
0.0
0.1
0.2
0.3
0
3
6
9
12
Time
Temperature
Baseline
−20C
−80C

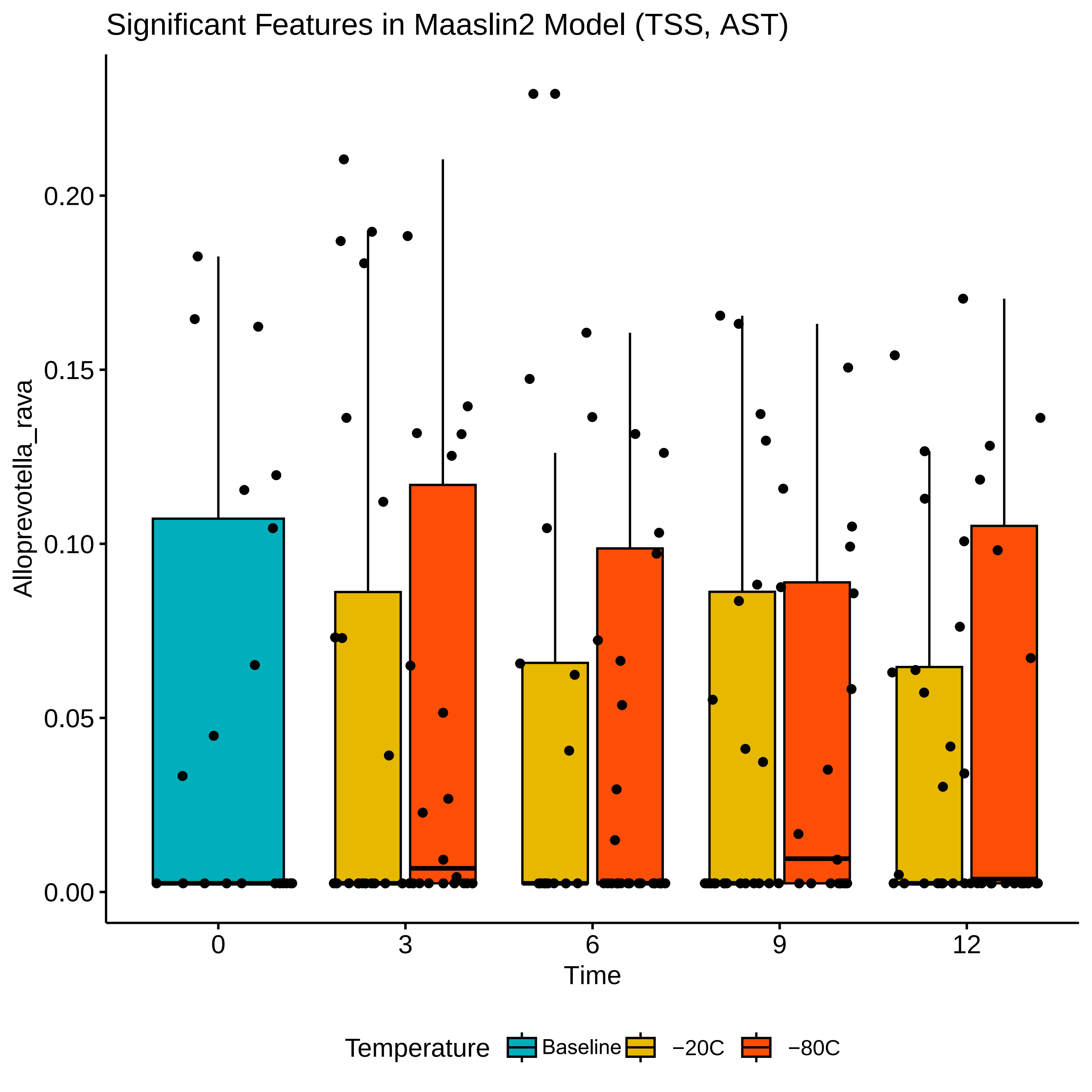
Significant Features in Maaslin2 Model (TSS, AST)
Alloprevotella_rava
0.00
0.05
0.10
0.15
0.20
0
3
6
9
12
Time
Temperature
Baseline
−20C
−80C

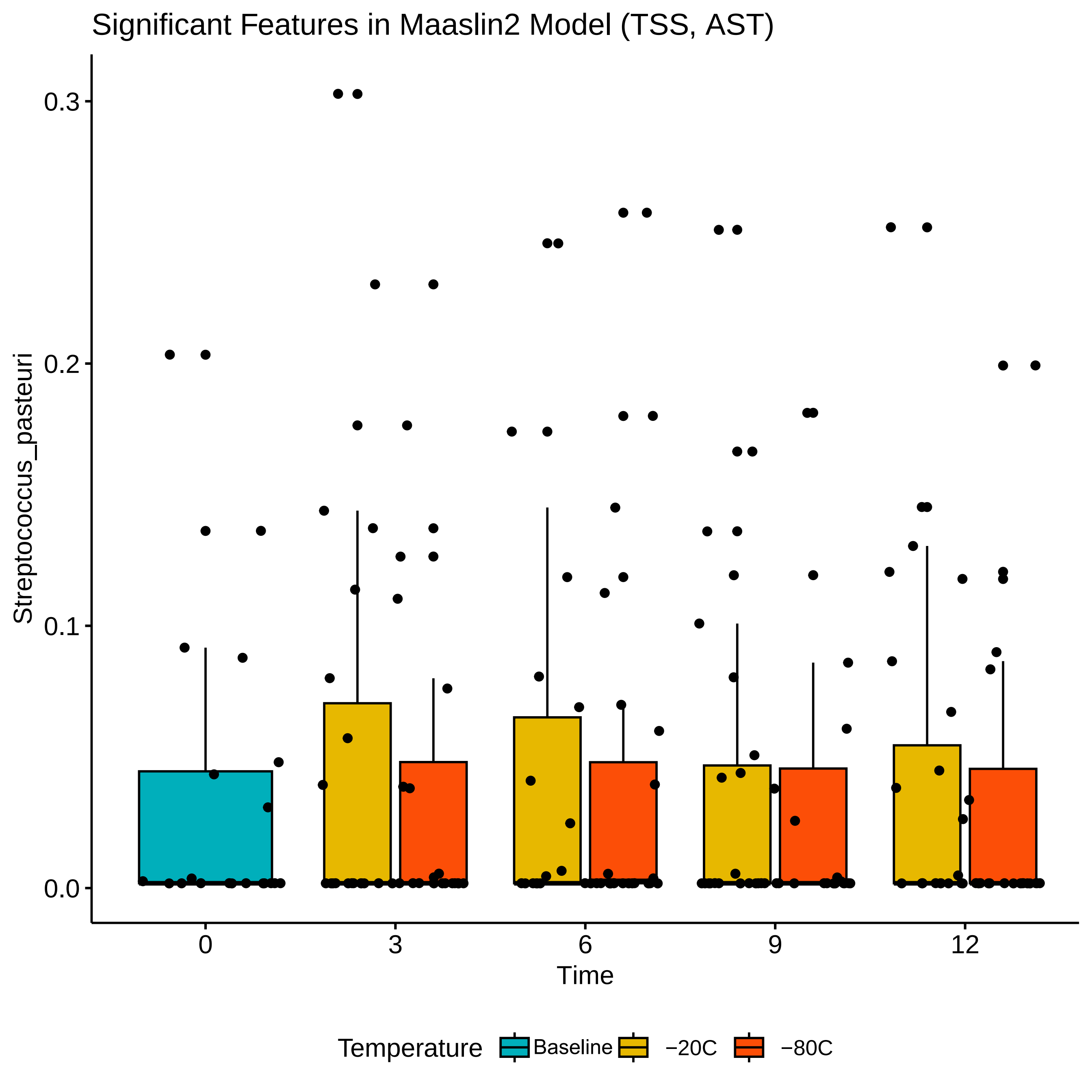
Significant Features in Maaslin2 Model (TSS, AST)
Streptococcus_pasteuri
0.0
0.1
0.2
0.3
0
3
6
9
12
Time
Temperature
Baseline
−20C
−80C

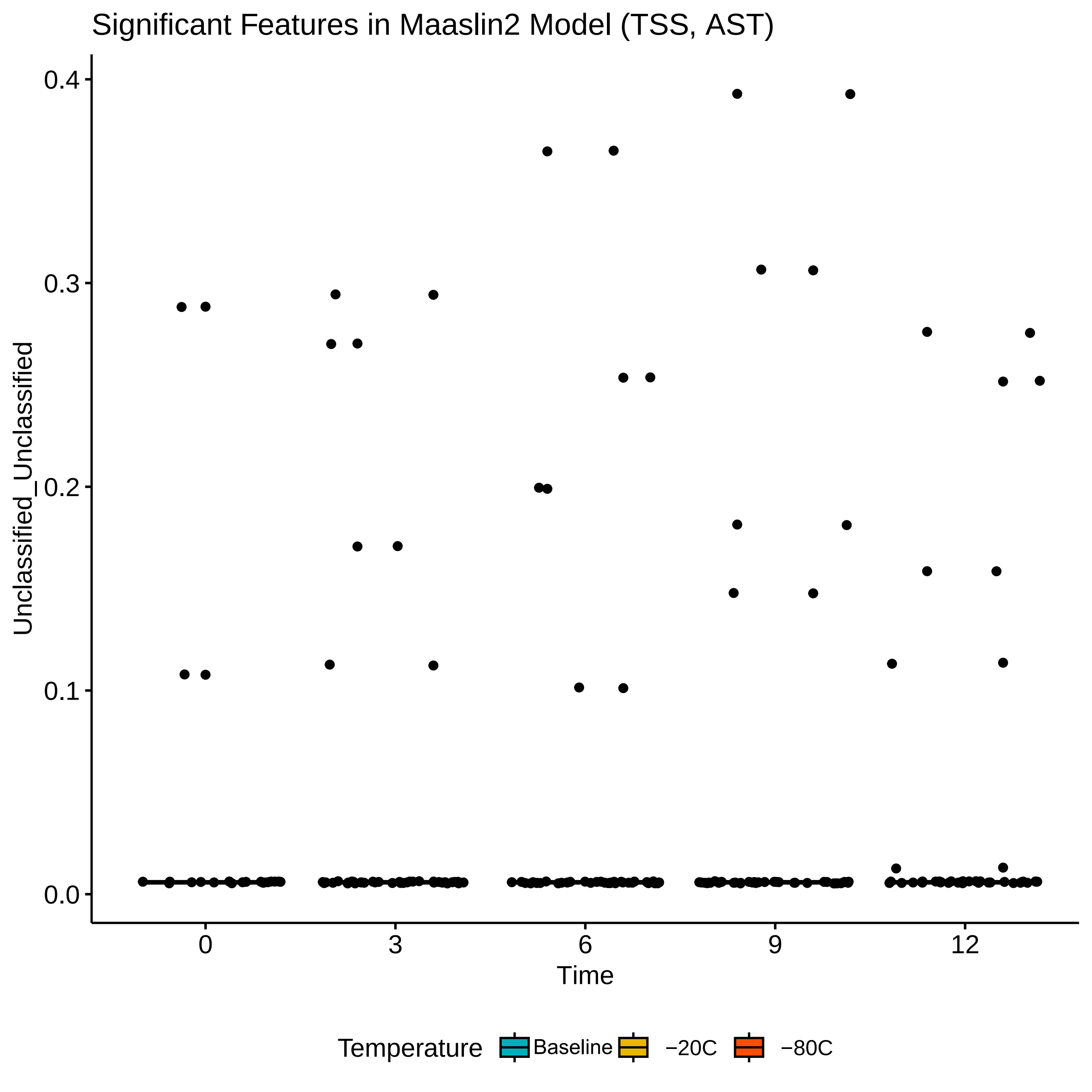
Significant Features in Maaslin2 Model (TSS, AST)
Unclassified_Unclassified
0.4
0.3
0.2
0.1
0.0
0
3
6
9
12
Time
Temperature
Baseline
−20C
−80C

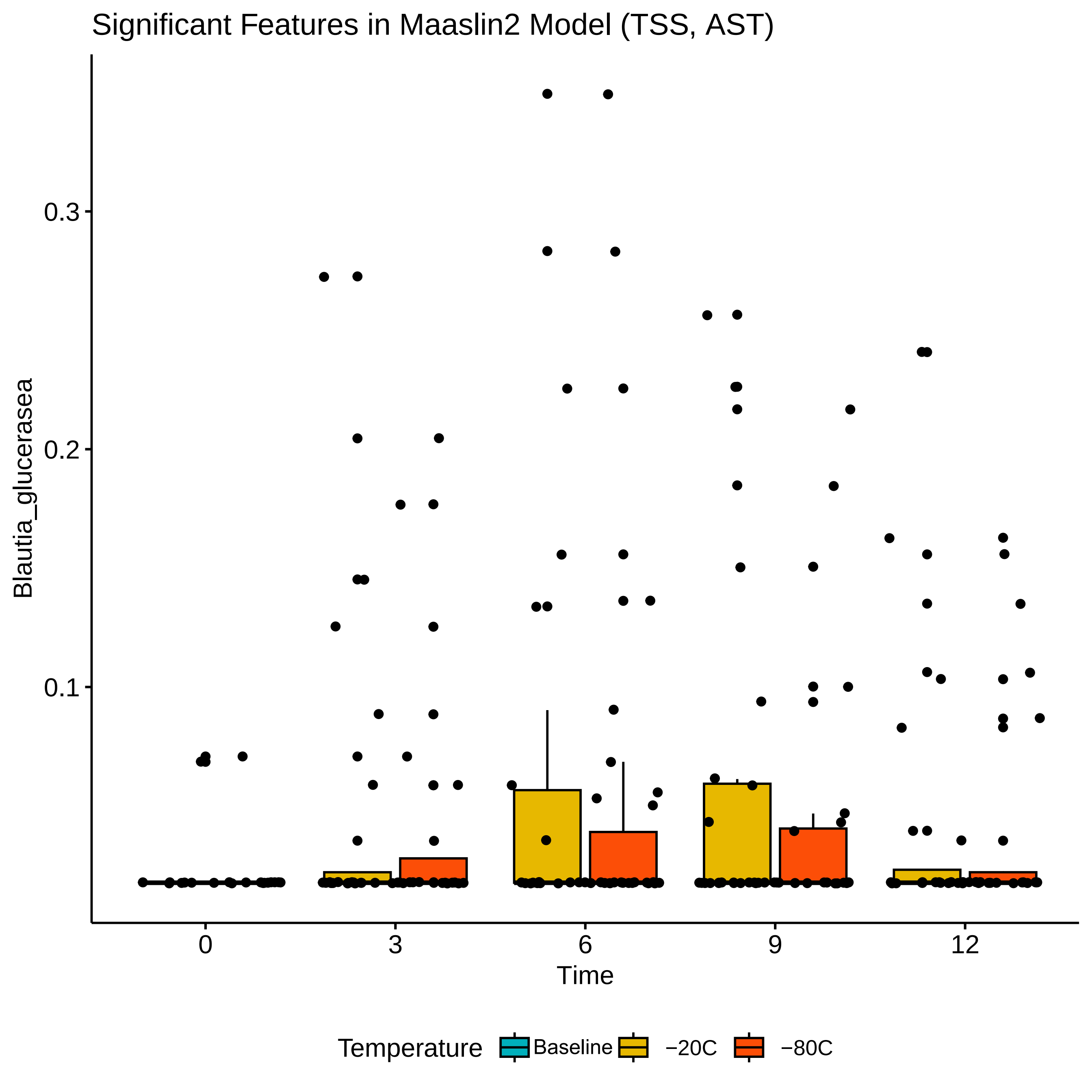
Significant Features in Maaslin2 Model (TSS, AST)
Blautia_glucerasea
0.3
0.2
0.1
0
3
6
9
12
Time
Temperature
Baseline
−20C
−80C

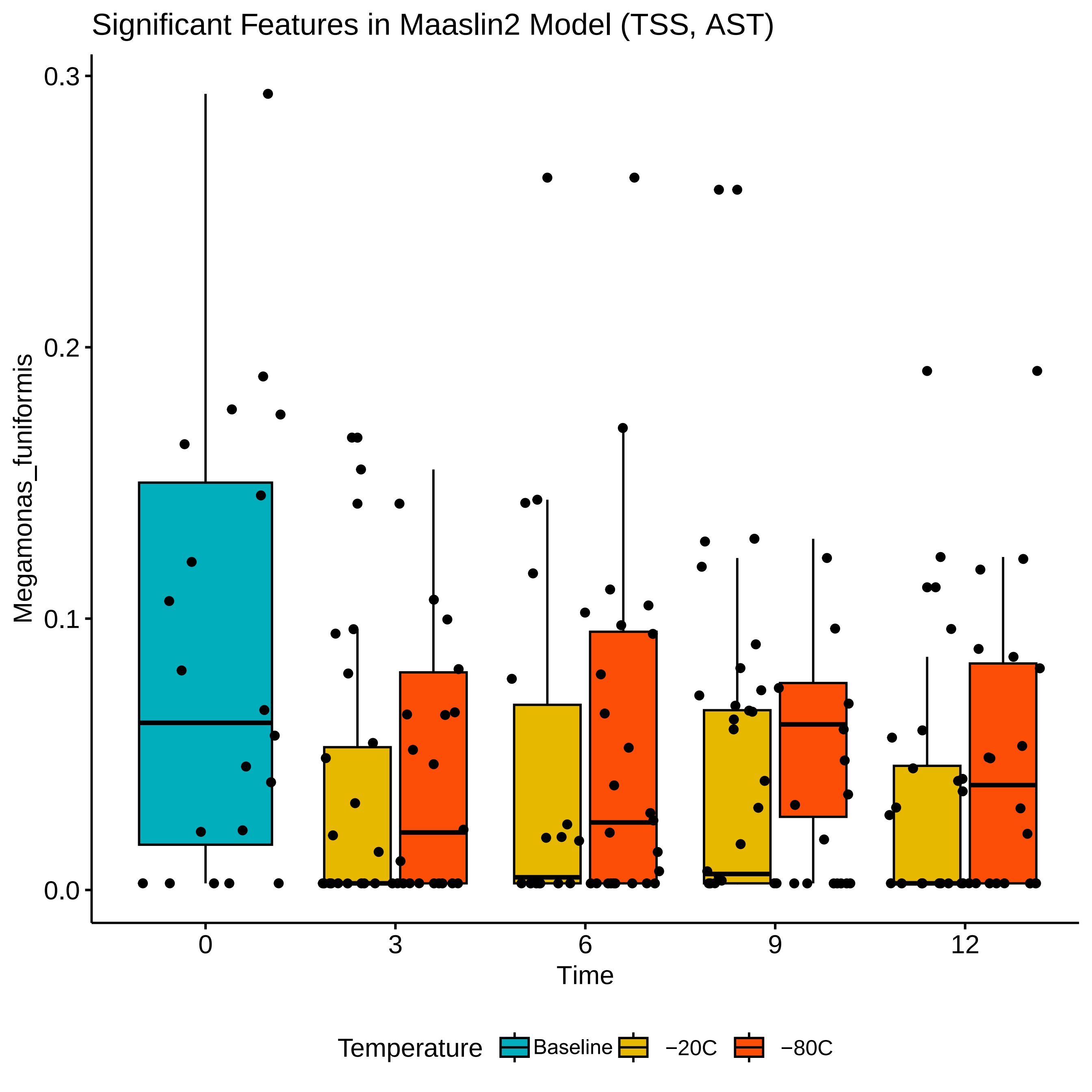

Significant Features in Maaslin2 Model (TSS, AST)
Megamonas_funiformis
0.3
0.2
0.1
0.0
0
3
6
9
12
Time
Temperature
Baseline
−20C
−80C

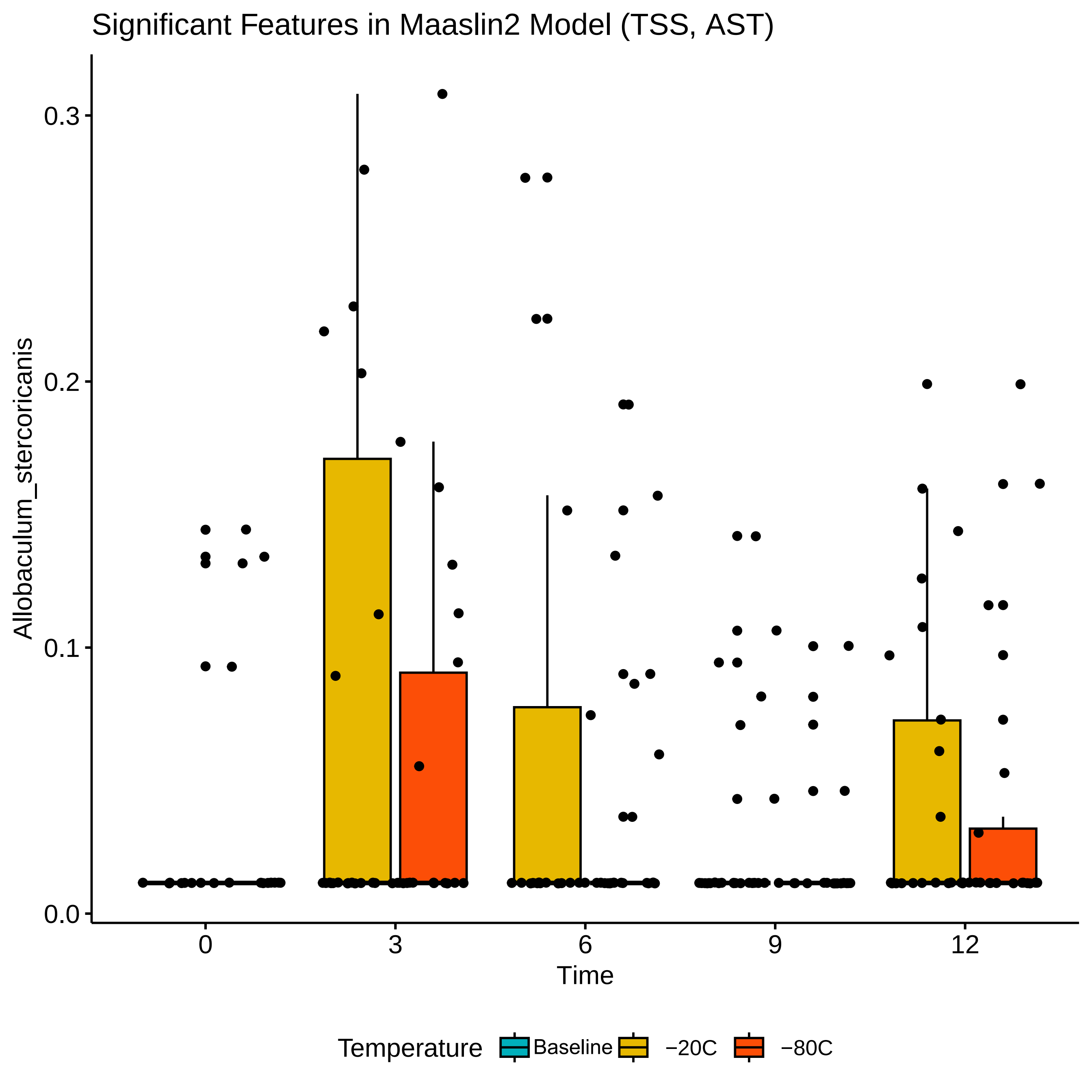

Significant Features in Maaslin2 Model (TSS, AST)
Allobaculum_stercoricanis
0.0
0.1
0.2
0.3
0
3
6
9
12
Time
Temperature
Baseline
−20C
−80C

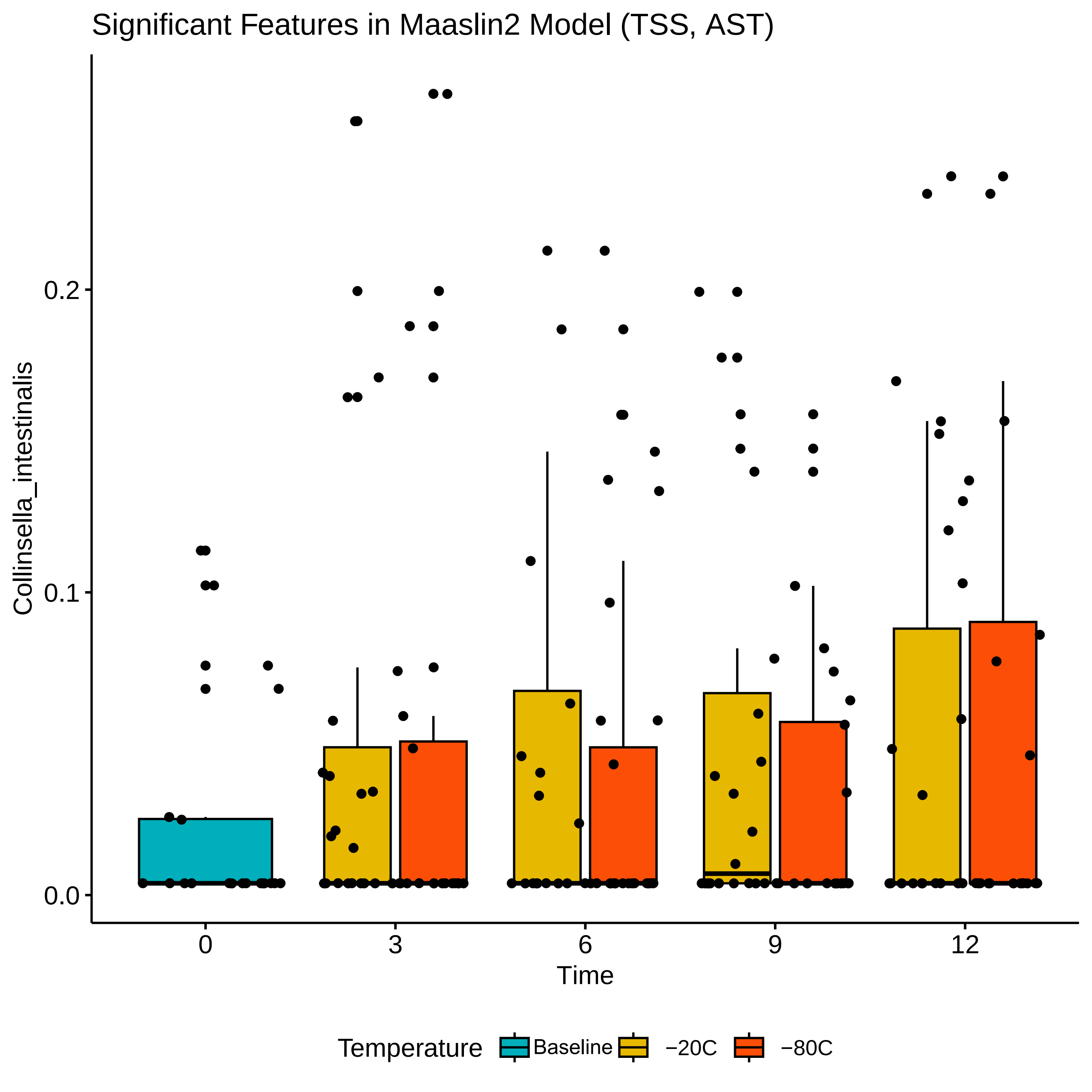
Significant Features in Maaslin2 Model (TSS, AST)
Collinsella_intestinalis
0.0
0.1
0.2
0
3
6
9
12
Time
Temperature
Baseline
−20C
−80C

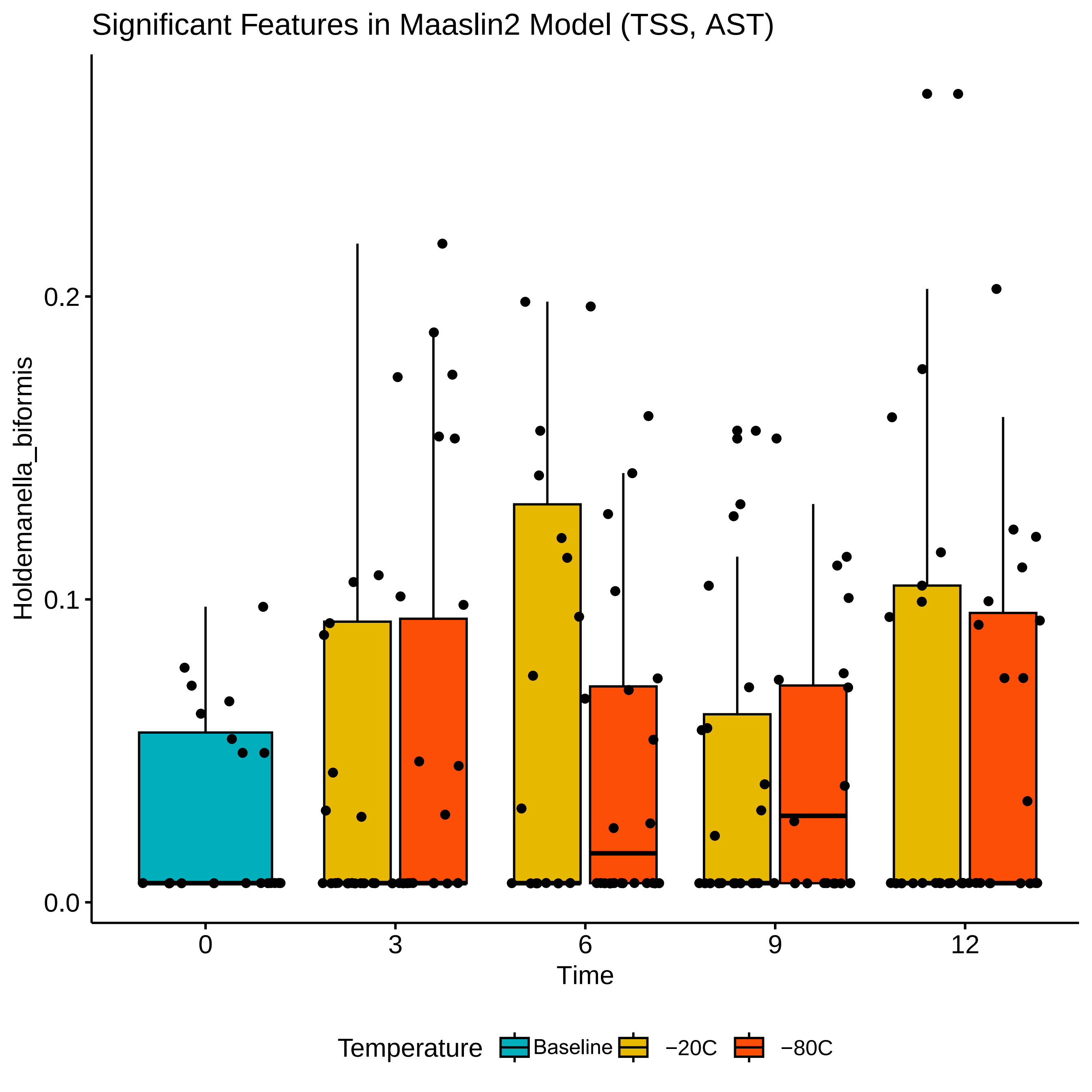

Significant Features in Maaslin2 Model (TSS, AST)
Holdemanella_biformis
0.0
0.1
0.2
0
3
6
9
12
Time
Temperature
Baseline
−20C
−80C

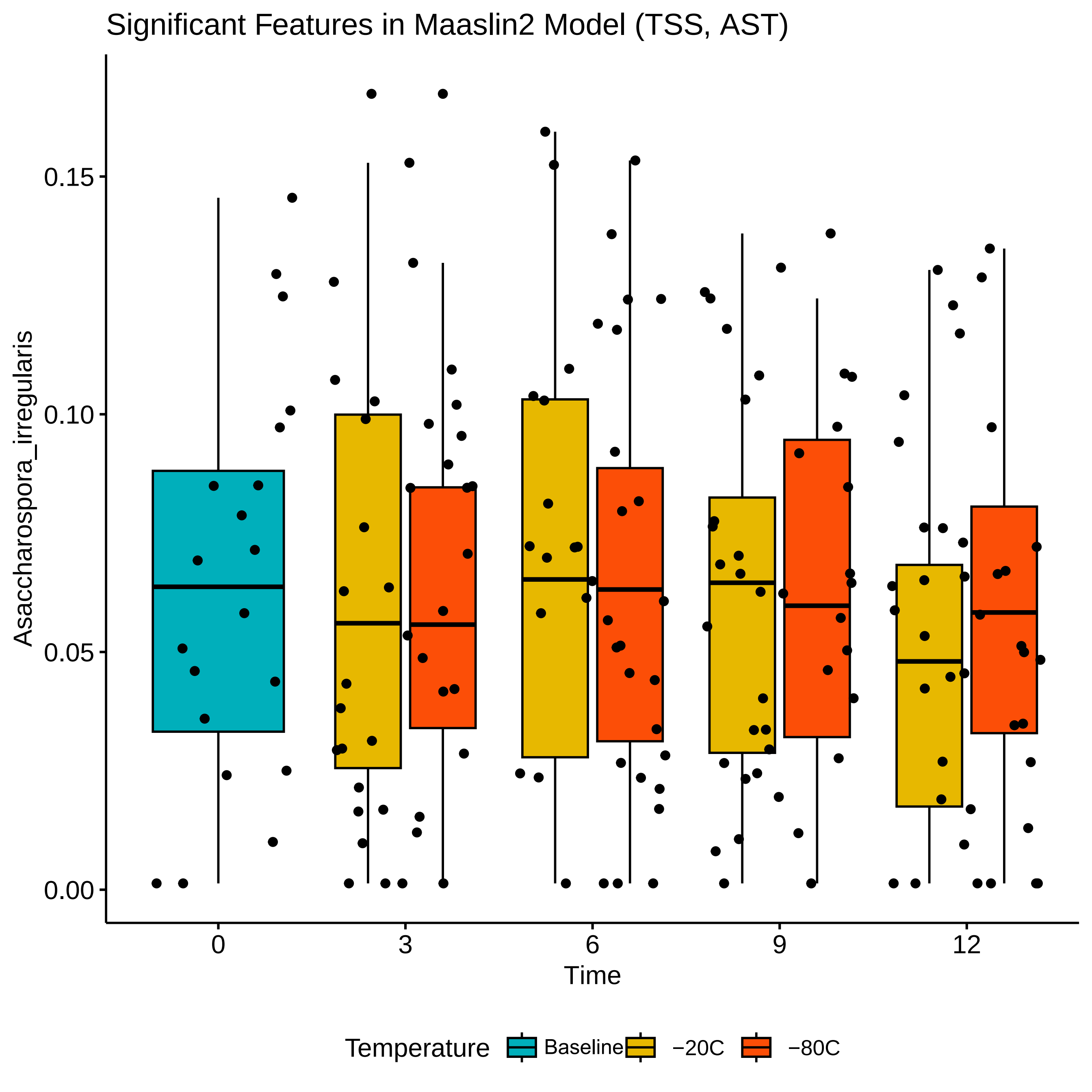

Significant Features in Maaslin2 Model (TSS, AST)
Asaccharospora_irregularis
0.00
0.05
0.10
0.15
0
3
6
9
12
Time
Temperature
Baseline
−20C
−80C

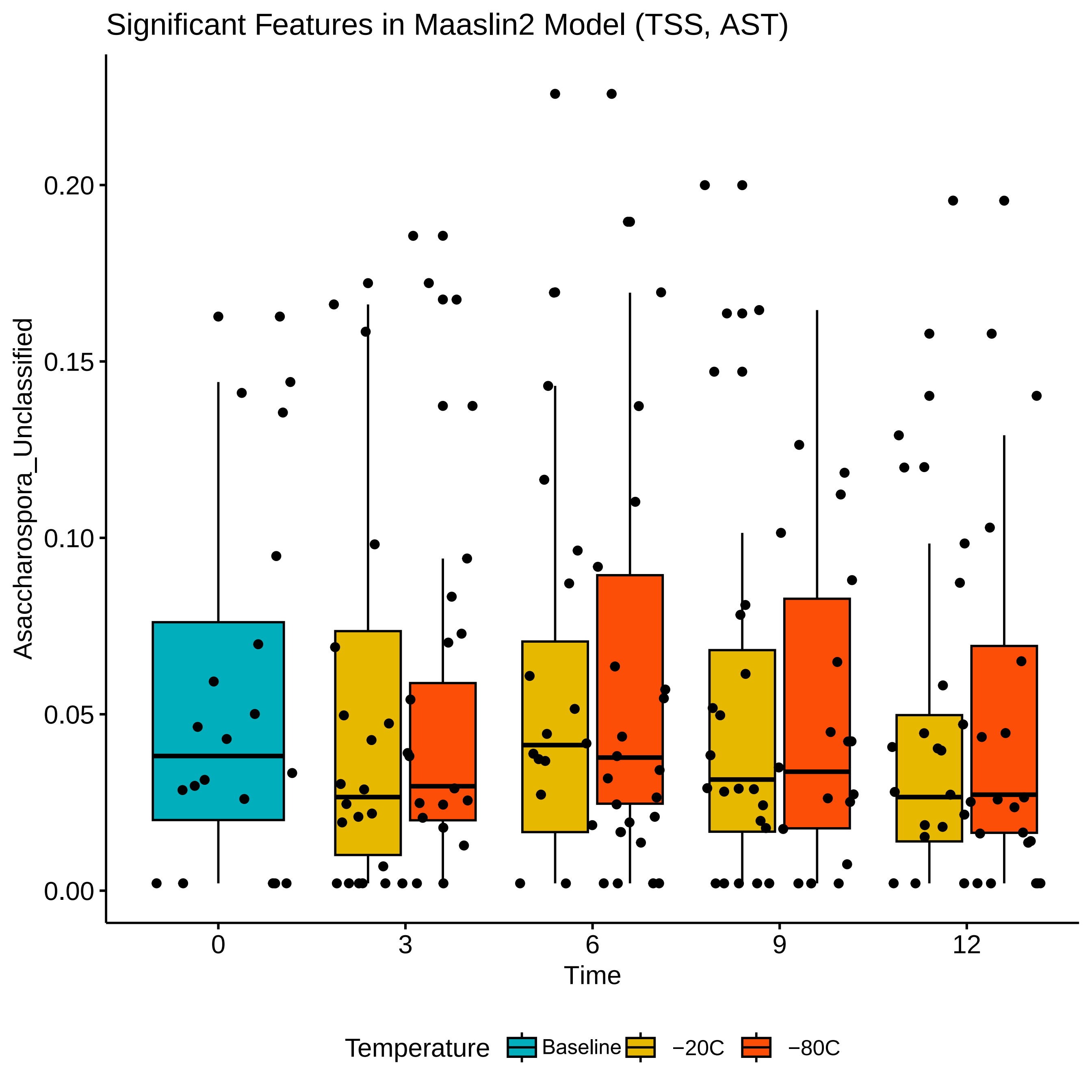
Significant Features in Maaslin2 Model (TSS, AST)
Asaccharospora_Unclassified
0.00
0.05
0.10
0.15
0.20
0
3
6
9
12
Time
Temperature
Baseline
−20C
−80C

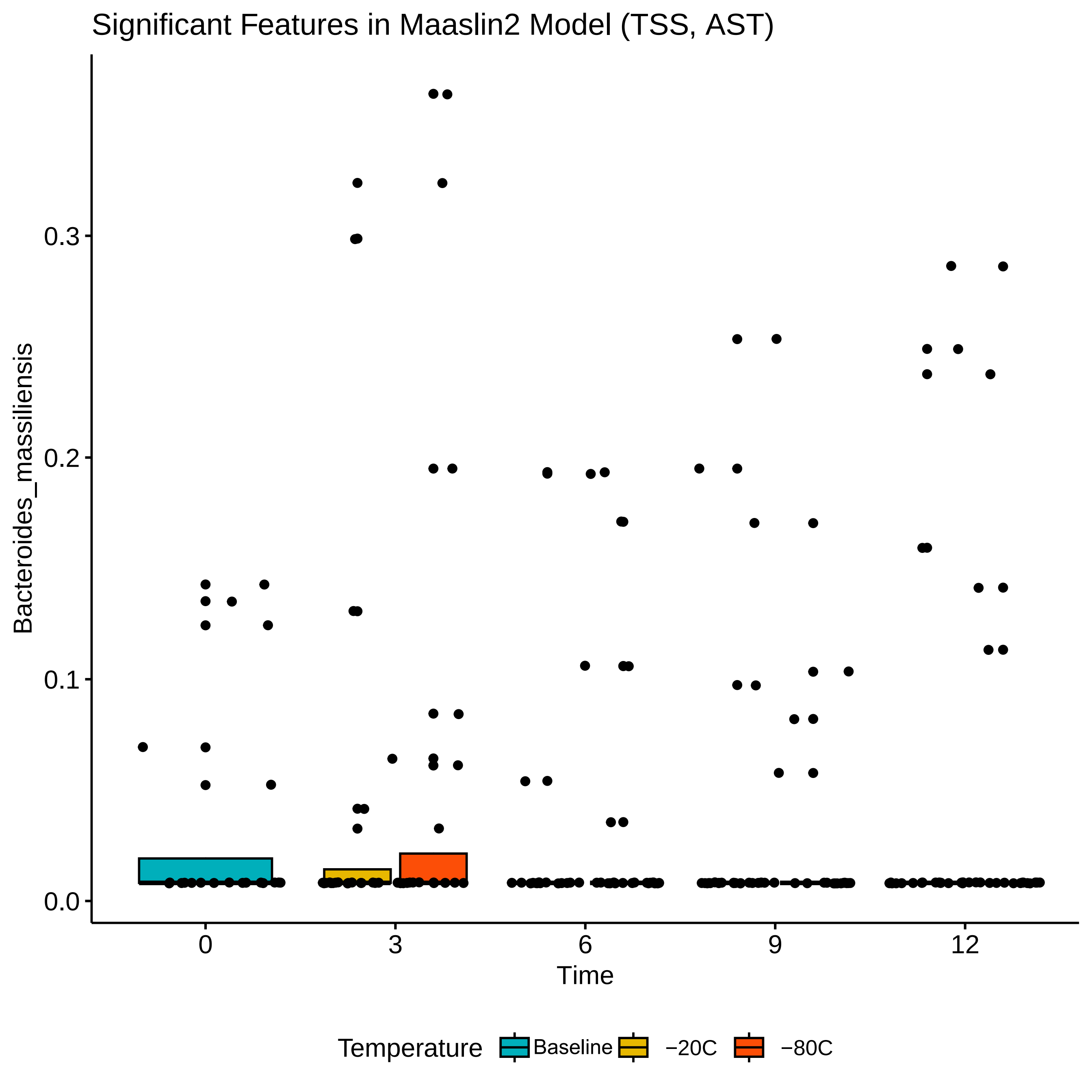

Significant Features in Maaslin2 Model (TSS, AST)
Bacteroides_massiliensis
0.0
0.1
0.2
0.3
0
3
6
9
12
Time
Temperature
Baseline
−20C
−80C
