# Supplementary material for: Fecal microbiome composition and diversity of cryopreserved canine stool at different duration and storage conditions
Source: PLoS One. 2024 Feb 7;19(2):e0294730. doi: 10.1371/journal.pone.0294730 (PMC10849402; doi:10.1371/journal.pone.0294730)
Supplement: Attachment — Submitted filename: Response to Reviewers.docx [file pone.0294730.s004.docx]

Dear Editor and Reviewers,

We thank the Reviewers for their thoughtful critique and for the time spent on reviewing our manuscript and providing comments to improve it. In the revised manuscript we addressed Reviewer #3’s two comments and followed their suggestion for the first comment and indicated in the manuscript why we could not follow their second suggestion. Please see below our line-by-line responses. All manuscript changes were made using the Microsoft’s Track-Change function as instructed by the Editor.

Reviewer #3:

Line 50: Authors to update naming nomenclature for "Clostridum" to "Clostridioides" throughout the article and note that recurrent CDI-associated colitis is different from recurrent CDI.

Authors’ response: Per the Reviewer’s suggestion we changed "Clostridum" to "Clostridioides" throughout the manuscript.

Also, we have used the term “recurrent CDI-associated colitis” interchangeably with “recurrent CDI”. We see the Reviewer’s point that recurrent CDI-associated colitis is a colonic manifestation of recurrent CDI and because what we had previously referred to in the manuscript was recurrent CDI-associated colitis, in this revised manuscript we changed “recurrent CDI” to “recurrent CDI-associated colitis” throughout the manuscript.

Line 214: Microbial diversity and composition are largely influenced by diet, and the lack of diet log variation amongst these dogs had and environmental microbes from soil that they were exposed to was missing from the manuscript as a limitation to analysis. If such data is available, propose to cluster dogs by type of food diet and area they were exposed to for clustering in microbial composition and diversity rather than via breed to ascertain which composition is more stable by storage condition and duration.

Authors’ response: We did not collect those data so unfortunately, we cannot follow the Reviewer’s suggestion. We added the following sentence in lines 428-432 to highlight the Reviewer’s comment: “Lastly, we did not record the diet and geographical locations where the dogs resided. Both diet and soil bacteria from different geographic locations could have impacted the gut microbial community structure of the dogs. Including these factors in the multivariate analysis potentially could have revealed important information about their effect on the stability of cryopreserved stool.”
